# Immunogenetics: Genome-Wide Association of Non-Progressive HIV and Viral Load Control: *HLA* Genes and Beyond

**DOI:** 10.3389/fimmu.2013.00118

**Published:** 2013-05-27

**Authors:** Sophie Limou, Jean-François Zagury

**Affiliations:** ^1^Basic Science Program, Basic Research Laboratory, Frederick National Laboratory for Cancer ResearchFrederick, MD, USA; ^2^Chaire de Bioinformatique, Laboratoire Génomique Bioinformatique et Applications (EA 4627), Conservatoire National des Arts et MétiersParis, France

**Keywords:** genome-wide association study, SNP, HIV-1, viral control, long-term non-progression, chemokine receptors region, HLA

## Abstract

Very early after the identification of the human immunodeficiency virus (HIV), host genetics factors were anticipated to play a role in viral control and disease progression. As early as the mid-1990s, candidate gene studies demonstrated a central role for the chemokine co-receptor/ligand (e.g., CCR5) and human leukocyte antigen (HLA) systems. In the last decade, the advent of genome-wide arrays opened a new era for unbiased genetic exploration of the genome and brought big expectations for the identification of new unexpected genes and pathways involved in HIV/AIDS. More than 15 genome-wide association studies targeting various HIV-linked phenotypes have been published since 2007. Surprisingly, only the two HIV-chemokine co-receptors and *HLA* loci have exhibited consistent and reproducible statistically significant genetic associations. In this chapter, we will review the findings from the genome-wide studies focusing especially on non-progressive and HIV control phenotypes, and discuss the current perspectives.

## Introduction

Shortly after the discovery of human immunodeficiency virus (HIV)-1 as the etiologic agent for AIDS, it became apparent that this infection was exhibiting a considerable phenotypic heterogeneity at different levels:

(i) Virus acquisition: some individuals, named highly exposed HIV-seronegatives, remain uninfected even after repeated exposure to the virus (e.g., discordant couples, persons exposed to HIV-contaminated blood during transfusion, or sex workers) (Ludlam et al., 1985; Langlade-Demoyen et al., 1994; Fowke et al., 1996).
(ii) Disease progression: some infected individuals, named long-term non-progressors (LTNP), are able to immunologically control the infection for a long period of time (≥8 years) without any antiretroviral therapy by maintaining high CD4^+^ T-cells levels; other individuals, named the elite controllers, are able to successfully suppress the virus to very low or even undetectable level; in contrast, rapid progressors succumb to AIDS in absence of treatment after only a few years of infection (Pantaleo and Fauci, 1996; Saksena et al., 2007).
(iii) Response to treatment: some individuals may develop severe side effects in response to certain antiretroviral drugs (Hetherington et al., 2002; Mallal et al., 2002).

This phenotypic heterogeneity may be attributed to a complex interplay between viral, environmental, and host genetic factors, and *de facto*, host genes have been extensively explored since the 1990s using candidate gene association studies. The first discovery involved a 32 bp deletion in *CCR5* (*CCR5-Δ32*) coding for a truncated and non-functional protein version of the major HIV-1 entry co-receptor. Homozygous Δ32/Δ32 individuals no longer express the co-receptor on the cell surface, which affords these fortunate individuals near complete protection from HIV-1 infection, while Δ32/+ heterozygous individuals exhibit a decreased cell surface protein expression and a delayed AIDS progression (Dean et al., 1996; Liu et al., 1996; Samson et al., 1996). This critical finding has fostered the development of a new antiretroviral class of inhibitors, which are designed to interfere with the HIV-CCR5 interaction, and two of them are already included in current treatment regimens (Henrich and Kuritzkes, 2013). This successful story of translation to bedside reveals the power of leverage for host genetics in the fight against the AIDS epidemic.

Additional genetic associations were identified with HIV clinical course and highlighted notably the importance of the human leukocyte antigen (HLA) alleles (e.g., B*27, B*35, B*57), of several immunity-related genes (e.g., *KIR*, *IL10*, *IFNγ*), and of host genes encoding HIV restriction factors (e.g., *CCL5*, *APOBEC3G*, *CUL5*). While *HLA* associations were consistently replicated across studies (Kaslow et al., 1996; Carrington et al., 1999; Hendel et al., 1999), others are still controversial. The functional interpretation for most of these variants is yet to be discovered, but the detailed account of each candidate gene is beyond the scope of this article, and has been covered by previous enlightening reviews (Fellay, 2009; An and Winkler, 2010). Overall, most of the candidate gene associations displayed small to modest effect sizes, and combined all together account for a small fraction of the phenotype variability (O’Brien and Nelson, 2004).

## Key Advance: Genome-Wide Association Studies

In this context, the advent of a series of technological and scientific progresses enabling the exploration of nearly the entire human genome without any *a priori* motivated the scientific community to pursue the discovery of host genes contributing to HIV-related outcomes. To a large extent, genome-wide association studies (GWAS) were a direct consequence of the achievement of International HapMap Consortium (2003), which compiled common human genetic variations across several continental populations. Due to the high correlation or linkage disequilibrium (LD) existing between genetic variants or SNPs, the complexity of the entire human genome can be recapitulated by a smaller subset of informative SNPs, called tagSNPs. Genotyping this subset of tagSNPs offered an unbiased exploration of most of the common genetic variation of the human genome, opening up opportunities for novel discoveries. At the same time, accurate high throughput chip technologies were developed to genotype cost-effectively, rapidly, and almost effortless hundreds of thousands to millions of SNPs in parallel (Gunderson et al., 2005), which combined with knowledge from the HapMap project, led to a highly prolific era for genetic interrogation of complex diseases.

Nevertheless, this global hypothesis-free approach also carries limitations:

(1) Genome-wide association studies requires a very large number of subjects with homogeneous ethnicity and phenotype in order to get enough power to overcome the cost of multiple comparisons and of very stringent genome-wide significance threshold. Power is the key in complex diseases: in contrast to Mendelian diseases caused by a single gene, complex diseases are often caused by a multitude of variants with small to medium effect size (odds-ratio, OR). To identify these variants while respecting the strict statistical expectations of GWAS, a very large number of samples is thus necessary. As an illustration of this limitation, a GWAS exploring adult height in about 200,000 Europeans identified over 180 loci with OR ranging from 1.02 to 1.3, which explain only 10% of the variance in adult height (Lango Allen et al., 2010).
(2) The first generation of genotyping arrays only covered common variations of the human genome (with a frequency ≥5%). As a consequence, less frequent SNPs, indels, and structural variants that may be involved in the pathogenesis could not be identified by this approach.
(3) Genotyping arrays targeting representative tagSNPs, the identification of the true causal variant is unlikely and often requires fine mapping.
(4) Africa contains the oldest populations in the world and has more generational time for recombination events to occur. African populations therefore exhibit a higher genetic diversity (larger number of polymorphisms) and a lower LD level. The genome-wide capture of common variation is thus currently less effective in African populations compared to European or Asian populations.

The scientific community embraced GWAS as a method to unravel the mysteries of genetics underlying complex diseases and traits. These studies proliferated to such a degree that a dedicated website listing all the GWAS findings was created (http://www.genome.gov/26525384). This enthusiasm extended to the HIV field with the publication of over 15 GWAS since the first article in 2007. In the next sections, we will review the main GWAS findings by emphasizing especially the differences in gene associations among different HIV-linked phenotypes: viral load control, AIDS progression, and HIV acquisition.

## GWAS Focusing on Viral Load Control

**Figure 1:**
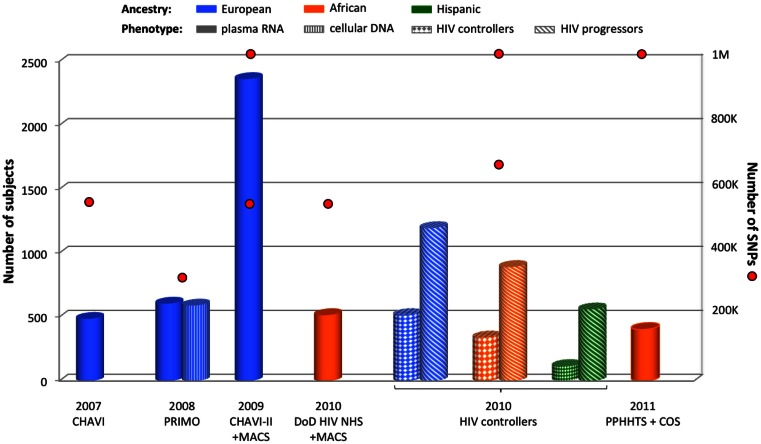
**Summary of GWAS design focusing on viral load control**. The histogram presents the number of subjects and red circles display the number of SNPs covered by the genotyping arrays. Two red circles are symbolized for one cohort when individuals were genotyped using two sets of arrays. Individual ancestry is color-coded: blue for European, orange for African, and green for Hispanic. The pattern of each cylinder illustrates the phenotype of interest: plain color for plasma RNA, vertical stripes for cellular DNA, solid diamonds for HIV controllers, and diagonal stripes for HIV progressors.

**Figure 2:**
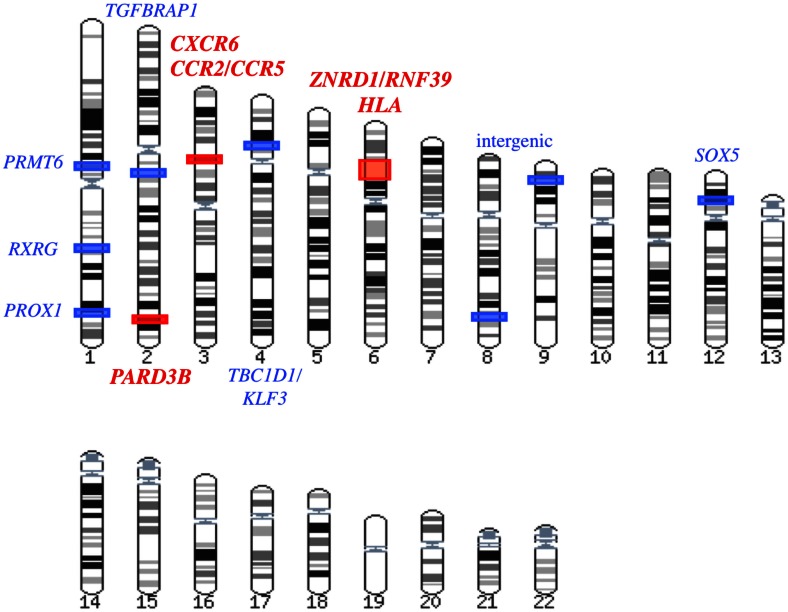
**Summary of the GWAS results associated with AIDS progression and/or viral load control**. In this karyotype, the statistical significance of the GWAS hits is color-coded: red for genome-wide significant loci and blue for candidate loci that did not reach genome-wide significance but were considered noteworthy by the authors of the study.

The first GWAS focusing on HIV-related outcomes was published in 2007 for the Euro-CHAVI cohort (Fellay et al., 2007). This international consortium assessed genetic association with stable plasma RNA viral load during the asymptomatic stage of HIV infection for 486 European HIV-infected individuals (Figure 1). The exploration of 550,000 tagSNPs revealed only two independent signals respecting the genome-wide significance threshold (Figure 2): rs2395029 (*P* = 9.36 × 10^−12^) and rs9264942 (*P* = 3.77 × 10^−9^). Both polymorphisms are located in the highly polymorphic chromosome 6 *HLA* region: rs2395029 is located in the *HCP5* gene and is in near complete LD with the previously known HLA-B*5701 HIV-1 protective allele; and rs9264942 is located 35 kb upstream *HLA-C*. For both associations, causal variants were sought and functional exploration was undertaken (see below).

To increase the power of this first study, additional European subjects were recruited from the Euro-CHAVI consortium and from the USA-based MACS cohort (*n* = 2,362 including the previous 486 participants) (Fellay et al., 2009). These individuals were genotyped on the Illumina 550K or 1 M platforms (Figure 1), and the analysis of RNA viral load at set point confirmed the two previous *HLA* signals. Numerous significant variants were also identified in the *HLA* region, but after correcting the analysis for rs2395029 and rs9264942 effects, only four independent SNPs remained: *C6orf12* rs259919 upstream *ZNRD1*, rs9468692 correlated with a missense *TRIM10* variant, rs9266409 downstream *HLA-B*, and the missense rs8192591 *NOTCH4* SNP. In addition, genotyping of SNPs previously associated with viral load control in candidate gene studies confirmed the role of the chemokine receptors variants: *CCR5-Δ32*, *CCR5-P1*, and *CCR2-64I*.

Plasma HIV-RNA was assessed in 605 seroconverters from the French PRIMO cohort during the first 6 months after HIV infection, as well as cellular HIV-DNA (reflecting the HIV reservoir) in a subset of 590 subjects (Dalmasso et al., 2008) (Figure 1). Although no signal passed the Bonferroni threshold for genome-wide significance, the HIV-RNA GWAS still pointed out the importance of the *HLA* region and *HCP5* rs2395029 for viral load control. It also suggested a potential chromosome 4 signal for rs11725412, located between *TBC1D1* and *KLF3* genes, which was supported by an enrichment of the protective allele in a group of 45 long-term HIV controllers (HIV-RNA <400 copies/mL after 10 years of infection). In the GWAS focusing on cellular HIV-DNA phenotype, the top signal was again *HCP5* rs2395029 (*P* = 6.72 × 10^−7^, not reaching genome-wide significance), thus revealing the importance of the *HLA* region for both HIV replication and HIV reservoir.

Similarly to the Euro-CHAVI cohort design, 515 HIV-infected African-Americans with stable plasma RNA viral load at set point were recruited from the Department of Defense HIV Natural History Study (DoD HIV NHS) and the MACS (Figure 1) (Pelak et al., 2010). The GWAS did not reveal any genome-wide significant signal, but supplemental *HLA* typing revealed LD between the top SNP, rs2523608, and HLA-B*5703. This *HLA* allele was significantly associated with viral load control (*P* = 5.6 × 10^−10^), and explained 10% of viral load variance at set point in this cohort. Therefore, in a similar fashion to what has been observed for individuals of European ancestry, this first GWAS in an African ancestry group emphasized the main role played by HLA-B*57 alleles for viral load control.

The international HIV controllers study explored the genetic determinants of HIV-1 control by comparing HIV controllers (cases defined by a stable plasma HIV-RNA <2,000 copies/mL during at least 1 year without any treatment) with untreated chronically infected individuals (controls) with different ancestries: 516 cases vs. 1,196 controls for Europeans, 341 cases vs. 892 controls for African-Americans, and 117 cases vs. 560 controls for Hispanics (Figure 1) (International HIV Controllers Study et al., 2010). The GWAS in European individuals confirmed the two previously known *HLA* SNPs and revealed two additional independent *HLA* SNPs: the −35 kb *HLA-C* rs9264942 SNP, the *HCP5* rs2395029, rs4418214 close to *MICA*, and the intronic *PSORS1C3* rs3131018. The GWAS in African-Americans identified four different independent *HLA* SNPs: the previously known HLA-B*5703/rs2523608, rs2255221, rs2523590, and rs9262632. The Hispanic GWAS was performed on a much smaller sample size and no signal reached genome-wide significance. However, the strongest signals were also located within the *HLA* region.

In a study focusing on HIV-1 serodiscordant heterosexual couples from East and Southern Africa (the Partners in Prevention HSV/HIV Transmission Study, PPHHTS) and from South Africa and Uganda (the Couples Observational Study, COS), a subset of 403 African seropositive individuals was available for analysis with stable plasma HIV-RNA at set point (Figure 1) (Lingappa et al., 2011). This GWAS did not reveal any genome-wide significant SNP.

Overall, these various GWAS identified the *HLA* region as the primary driver of viral control in individuals with European or African ancestry.

## GWAS Focusing on AIDS Disease Progression

**Figure 3:**
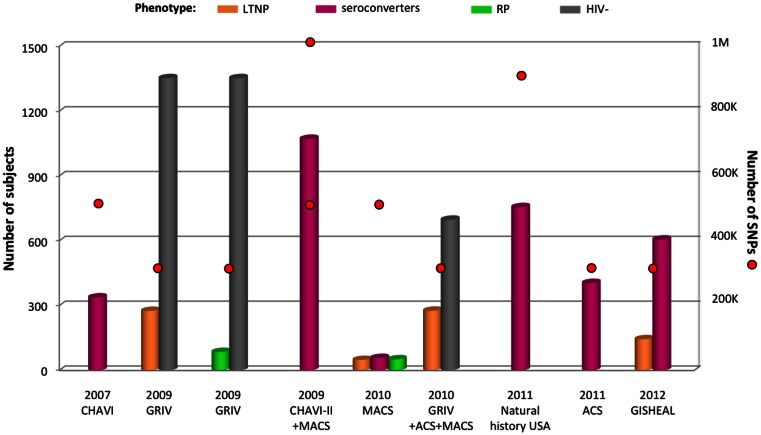
**Summary of GWAS design focusing on disease progression**. The histogram presents the number of subjects and red circles display the number of SNPs covered by the genotyping arrays. Two red circles are symbolized for one cohort when individuals were genotyped using two sets of arrays. All subjects are from European ancestry. The different phenotypes are color-coded: gold for long-term non-progressors (LTNP), pink for medium seroconverters, green for rapid progressors (RP), and gray for uninfected individuals. The natural history USA study comprises seroconverters from the following cohorts: ALIVE, DCG, HGDS, MACS, MHCS, and SFCC.

The Euro-CHAVI GWAS described above also explored host genetic factors associated with AIDS progression on a subset of 337 individuals from the Euro-CHAVI cohort (Figure 3) (Fellay et al., 2007). The progression phenotype was defined as the time to antiretroviral therapy initiation or to the drop of CD4^+^ T-cell count below 350/μL. A set of SNPs in the chromosome 6 *ZNRD1/RNF39* locus approached the genome-wide significance threshold (Figure 2). Their effect on progression and viral load set point was demonstrated to be independent from both the *HCP5* and −35 kb *HLA-C* SNPs.

The first genome-wide explorations of cohorts specifically designed to concentrate on AIDS progression rather than on viral load control were performed in the French GRIV cohort of participants with extreme progression profiles (Figure 3) (Le Clerc et al., 2009; Limou et al., 2009). GRIV patients were enrolled based on plasma CD4^+^ T-cell count, a major AIDS progression predictor in untreated patients. The 275 GRIV LTNP, defined by a CD4^+^ T-cell count above 500/μL for more than 8 years after HIV infection in absence of antiretroviral therapy, were compared to 1,352 HIV-1 seronegative individuals. The strongest signal associated with long-term non-progression was obtained for *HCP5* rs2395029 (*P* = 6.79 × 10^−10^). As illustrated by this result, host factors controlling viral load are likely to be correlated with stable high CD4^+^ T-cell count. Likewise, this explains the abundance of *HLA* SNPs (46 out of the 50 top signals) in the meta-analysis between the GRIV LTNP and the Euro-CHAVI HIV-RNA GWAS. These results emphasize the critical role of *HLA* both for early viral control and long-term non-progression. In addition, comparable to the Euro-CHAVI study, signals approaching genome-wide significance were identified independently from *HCP5* rs2395029 for *ZNRD1/RNF39* SNPs.

The only GWAS addressing rapid progression to date was performed by comparing the GRIV rapid progressors, defined by a drop of CD4^+^ T-cell count below 300/μL within 3 years after the last seronegative test, with 1,352 seronegative controls (Figure 3) (Le Clerc et al., 2009). The limited sample size for rapid progressors (*n* = 85) was not powered to detect variants respecting the genome-wide significance threshold. However, the use of an alternative statistical strategy, termed the false-discovery rate (Benjamini and Hochberg, 1995), revealed six independent loci potentially associated with rapid progression: *PRMT6* and *RXRG* (chr.1), *SOX5* (chr.12), *TGFBRAP1* (chr.2), and two intergenic loci (chr.8 and chr.9). These four candidate genes were biologically relevant for HIV/AIDS pathogenesis and notably emphasized a potential key role for the TGFβ pathway and for HIV replication control for progression. Nonetheless, the absence of a replication cohort enriched in rapid progressors is a severe limitation for further investigation of this unique progression phenotype.

Similarly to the first GWAS on Euro-CHAVI, the extended Euro-CHAVI and MACS cohort study assessed the progression phenotype for a subset of 1,071 subjects (Figure 3) (Fellay et al., 2009). This analysis confirmed the major role of *HCP5* rs2395029 and −35 kb *HLA-C* rs9264942 both for viral load control and disease progression. Due to increased power, the *ZNRD1/RNF39* candidate locus was for the first time significantly associated with AIDS progression (*P* = 1.8 × 10^−8^).

The *HCP5* and −35 kb *HLA-C* associations with disease progression are mainly driven by their influence on early viral load control: both *P-*values are much weaker if viral load is considered as a covariate in the statistical analysis of the extended Euro-CHAVI and MACS cohort (*P* < 10^−3^). On the other hand, *ZNRD1/RNF39* association is largely independent of viremia, suggesting a different mechanism of protection (Fellay et al., 2009). Similarly in the GRIV LTNP cohort, viral load was significantly lower in LTNP carrying the protective *HCP5* rs2395029-G allele, while none of the *ZNRD1/RNF39* polymorphisms seemed to correlate with viral load (Limou et al., 2009). In order to identify additional host factors impacting long-term non-progression without necessarily controlling viral load at very low level, the GRIV LTNP GWAS data were re-analyzed after excluding the elite controllers (with a viral load <100 copies/mL, Figure 3) (Limou et al., 2010). Individuals from the ACS and MACS cohorts respecting this specific non-progression phenotype were also included to increase power. The comparison of these 276 unique LTNP with 697 HIV-uninfected controls revealed a significant association for *CXCR6* rs2234358 (*P* = 2.1 × 10^−8^, Figure 2). This newly identified chromosome 3 signal was replicated in three independent European studies and shown to be independent from the *CCR2/CCR5* and *HLA* associations. CXCR6 is a minor HIV co-receptor (Deng et al., 1997) and is involved in inflammation (Landro et al., 2009) through the trafficking of effector T-cells (Kim et al., 2001) and the activation of NKT cells (Germanov et al., 2008). Interestingly, hepatic NK mice cells were shown to develop a specific adaptive immunity to HIV-1 antigens through a mechanism requiring the expression of CXCR6 (Paust et al., 2010).

Another GWAS was performed on a subset of subjects enriched with extreme progression profiles from the European-American MACS cohort: 51 rapid progressors vs. 57 medium progressors vs. 48 LTNP (Figure 3) (Herbeck et al., 2010). This GWAS was the first published study using the Affymetrix genotyping arrays, which carry a different set of SNPs (especially for the *HLA* region) compared to Illumina arrays. The top 25 signals were assessed for replication in an independent cohort of 590 seroconverters. No SNP reached genome-wide significance, but a potential signal was suggested for three related SNPs located 36 kb upstream the *PROX1* gene, encoding for a negative regulator of IFNγ (Wang et al., 2008).

Patients enrolled from six USA natural history cohorts (MACS, MHCS, DCG, HGDS, SFCC, and ALIVE) were genotyped using large coverage Affymetrix 6.0 arrays (Figure 3) (Troyer et al., 2011). Collectively, 755 European-American seroconverters were evaluated for association with progression to AIDS 1987. An intronic SNP in *PARD3B* reached genome-wide significance (*P* = 3.37 × 10^−9^), and several other *PARD3B* polymorphisms were among the top signals (Figure 2). One of these variants was replicated by comparing rapid progressors from the GRIV and ACS cohorts with uninfected controls (*P* = 0.025). PARD3B interacts with members of the SMAD family, which are involved in multiple signaling pathways and biological activities, and directly interact with HIV-1 (Ptak et al., 2008).

The Dutch seroconverters from ACS (*n* = 404) were tested for association with survival time to AIDS 1993-diagnosis or to AIDS-related death (Figure 3) (van Manen et al., 2011). The top 10 signals were assessed for replication in the independent GRIV cohort, and no genome-wide significant result was revealed. Potential associations were highlighted, but to date, none of them have been replicated. The *HCP5* rs2395029 SNP trended to association with time to AIDS 1993 or to AIDS-related death, but as previously reported, this effect was only marginal when the analysis was corrected for the effect of viral load at set point (van Manen et al., 2009).

Finally, the French-Italian GISHEAL cohort recruited 144 LTNP defined by a CD4^+^ T-cell count above 500/μL for more than 7 years after HIV infection in the absence of antiretroviral therapy (Figure 3) (Guergnon et al., 2012). The GWAS comparing these LTNP with the 605 seroconverters from the PRIMO cohort confirmed the importance of the *HLA* region for long-term non-progression (rs2395029, *P* = 1.61 × 10^−11^).

Surprisingly, GWAS exploring host factors involved in AIDS disease progression only focused on population of European ancestry. These studies confirmed that the *HLA* region is also strongly associated with long-term non-progression. The chromosome 6 *ZNRD1/RNF39* locus, the chemokine receptor region (*CCR2/CCR5* and *CXCR6*), and the *PARD3B* gene were also identified as key regulators of disease progression. Lastly, some biologically relevant candidates genes were suggested (such as *PRMT6*, *TGFBRAP1*, or *PROX1*), but they still require replication in an independent genetic study.

## GWAS Focusing on HIV Acquisition

**Figure 4:**
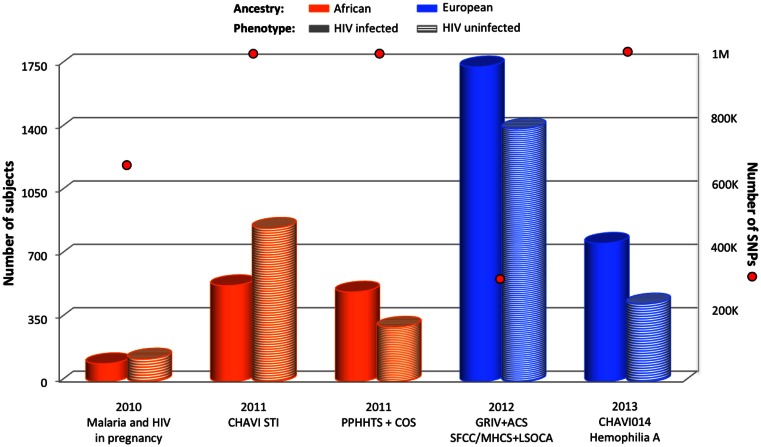
**Summary of GWAS design focusing on HIV acquisition**. The histogram presents the number of subjects and red circles display the number of SNPs covered by the genotyping arrays. Individual ancestry is color-coded: orange for African and blue for European. The pattern of each cylinder illustrates the phenotype of interest: plain color for HIV-infected, and horizontal stripes for HIV-uninfected.

The first GWAS exploring HIV acquisition focused on mother-to-child transmission in Malawi (Joubert et al., 2010). In this underpowered study, 100 HIV^+^ infants and 126 HIV^−^ infants from HIV^+^ mothers enrolled in the Malaria and HIV in Pregnancy prospective study were compared (Figure 4), but no genetic variant reached genome-wide significance.

A second larger GWAS performed on individuals from Malawi compared 531 HIV^+^ with 848 sexually active HIV^−^ recruited by CHAVI in sexually transmitted infection (STI) clinics (Figure 4) (Petrovski et al., 2011). It was assumed that the latter group was exposed to HIV-1 because of the very high prevalence of the infection among sexually active adults in Malawi. No genome-wide significant SNP was identified.

A third study performed on individuals from Africa explored the HIV acquisition in serodiscordant heterosexual couples (PPHHTS/COS study) (Lingappa et al., 2011). In this study, the risk of exposure to HIV was carefully scored, and 496 HIV^+^ individuals were compared to 302 HIV^−^ individuals (Figure 4). Nevertheless, this GWAS also failed to reveal any significant signal.

A first GWAS exploring HIV acquisition in individuals from European ancestry compared 764 HIV^+^ individuals from the GRIV and ACS cohorts with 1,073 HIV^−^ individuals (Figure 4) (Limou et al., 2012). The top signals were assessed for replication in two independent groups of European descent (MHCS-SFCC and LSOCA). The rs6996198-T allele (chr.8) was significantly associated with a protection from HIV-1 infection (*P* = 7.76 × 10^−8^). This protective allele was associated with a higher *CYP7B1* expression, a gene located 45 kb upstream the variant. CYP7B1 is involved in the steroid and oxysterol metabolism (Stiles et al., 2009), but also in immune control and regulation – notably in the IgA (Bauman et al., 2009) and proinflammatory cytokines (Dulos et al., 2005) production, and in the induction of programed cell death (Rusinol et al., 2004).

Recently, European hemophiliacs exposed to potentially HIV-contaminated blood through transfusion (CHAVI014) were investigated: 765 HIV^+^ individuals were compared to 430 HIV^−^ individuals who were not *CCR5-Δ32* homozygous (Figure 4) (Lane et al., 2013). The extensive screening of the genetic data did not reveal any significant association.

In summary, the GWAS focusing on HIV acquisition did not reveal any signal in African ancestry populations, and only one potential association with *CYP7B1* was identified in a European ancestry study.

## Causal Variant and Functional Exploration

Amongst the genetic variants highlighted through the GWAS (Figure 2), several were further explored to unravel the biological mechanism underlying the association, especially for the *HLA* region. The complex LD pattern in this region makes it very difficult to discriminate the causal variant(s), in particular in the context of a locus rich in immune-related genes that are all candidates of interest for the control of HIV replication and pathogenesis, and that have almost all been previously associated with various immune-related diseases.

For example, *HCP5* rs2395029 is in LD with HLA-B*5701, a securely identified *HLA* protective allele, but is also in LD with polymorphisms from *MICB*, *BAT1*, *TNF*, and *LTB*. MICB is a ligand for CD8^+^ T-cells and NK cells, which are both key players in the anti-HIV immune response. BAT1 is involved in splicing and RNA export (Reed and Hurt, 2002), and is a negative regulator of proinflammatory cytokines (Allcock et al., 2001). TNF is a key proinflammatory cytokine that has been widely investigated in HIV-1 infection (Kedzierska and Crowe, 2001). LTB is an inflammation modulator involved in the development of immune cells (Rennert et al., 1996). *HCP5* itself was considered as a candidate of interest: since it encodes a human endogenous retrovirus with sequence homology to HIV-1 *pol* (Kulski and Dawkins, 1999), it was hypothesized that it may act as an antisense RNA interfering with HIV replication. However, the two allelic forms do not impact HIV-1 replication *in vitro*, suggesting that *HCP5* rs2395029 is probably not the causal variant (Yoon et al., 2010). HLA-B*5701 was previously associated with low viremia and delayed onset of AIDS through candidate gene studies (Stephens, 2005). The potential function of this allele has been widely explored: notably, the presentation of specific HIV-1 epitopes able to initiate a strong cytotoxic T-lymphocyte response, and the TCR clonotype recognizing the HLA-peptide complex were largely emphasized (Klein et al., 1998; Migueles et al., 2000; Gillespie et al., 2002, 2006; Miura et al., 2009; Elahi et al., 2011; Chen et al., 2012; Kloverpris et al., 2012). Following this path, the international HIV controllers study confirmed the overwhelming importance of *HLA* alleles for viral control (B*5701, B*2705, B*14/C*0802, B*52, and A*25 as protective alleles, and B*35 and C*07 as risk alleles) and identified putative causal variants in the HLA-B peptide-binding groove (including residues at position 67, 70, and 97) in Europeans (International HIV Controllers Study et al., 2010). Similarly in African-Americans, they confirmed the importance of HLA-B*5703 and HLA-B*8101 alleles for viral control and identified the HLA-B groove residues 63, 97, and 116 as critical for the association (International HIV Controllers Study et al., 2010; McLaren et al., 2012a). Even though the mechanisms underpinning HIV-1 control and disease non-progression remain unclear, these coding variants are predicted to impact the conformation of the HLA-B groove and the HIV peptides that are presented, as well as the subsequent engagement of TCR or other receptors (such as the NK cell receptors). As suggested by the recent study by McLaren et al. (2012a), the role played by *HLA-B* on viral control does not rule out the implication of other polymorphisms in the *HLA* region, outside the *HLA-B* binding groove and possibly outside the class I *HLA* genes.

**Figure 5:**
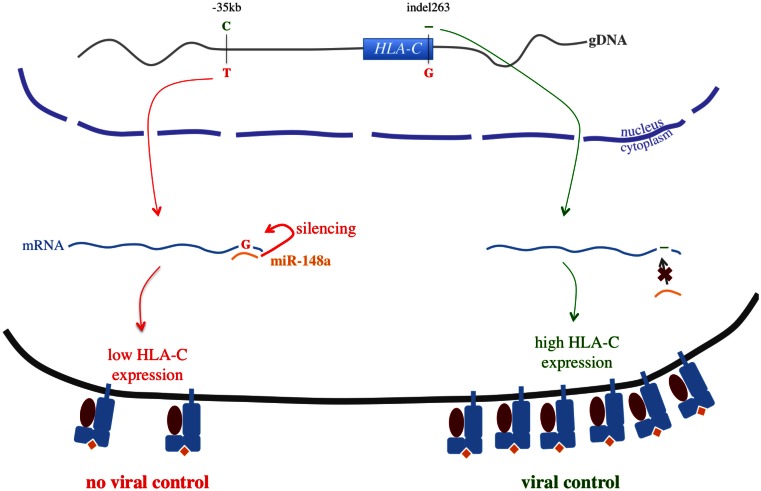
**−35 kb *HLA-C* functional story**. This picture illustrates the strong linkage disequilibrium between −35 kb *HLA-C* (rs9264942) and the 3′UTR indel263 (rs67384697): the −35 kb-C allele is strongly correlated with the deletion, when the −35 kb-T allele is strongly correlated with the insertion of a G nucleotide. If the mRNA carries the −35 kb-T/insertion haplotype, the human miR-148a can bind to the *HLA-C* 3′UTR and silence its expression, leading to a low HLA-C cell surface expression. However, if the mRNA carries the −35 kb-C/deletion haplotype, miR-148a cannot bind efficiently to the *HLA-C* 3′UTR anymore, and HLA-C cell surface expression is increased. This mechanism would mostly explain the association between the −35 kb *HLA-C* polymorphism and viral control.

The −35 kb (*HLA-C*)-C allele, associated with viral control, was correlated with higher *HLA-C* gene expression (Stranger et al., 2005) and higher HLA-C cell surface expression (Thomas et al., 2009). The signal was proposed to be the result of LD between the −35 kb-T allele and the HLA-C*07 allele, known to be associated with very low cell surface expression and more rapid disease progression (Corrah et al., 2011). Interestingly, the −35 kb-T allele was also demonstrated to be in LD with a polymorphism of the *HLA-C* 3′UTR (ins263 or rs67384697-G): this polymorphism results in the creation of a binding site for miR-148a causing the silencing of the *HLA-C* mRNA expression and a low HLA-C cell surface expression (Figure 5) (Kulkarni et al., 2011). This mechanism offers a highly plausible functional basis for the high HLA-C cell surface expression observed amongst HIV-infected individuals controlling the virus replication. However, since HLA-C expression occurs as a continuous gradient rather than a bimodal pattern that would be expected if miR-148a were the sole regulation pathway, additional unknown factors are likely to affect HLA-C expression.

Finally, a set of chromosome 6 SNPs in the *ZNRD1*/*RNF39* locus was associated with AIDS progression independently from the *HCP5* rs2395029 and −35 kb *HLA-C* signals. *ZNRD1* and *RNF39* encode for an RNA polymerase I subunit and for an unknown ring finger protein, respectively. *ZNRD1* expression was significantly correlated to several SNPs associated with CD4^+^ T-cell depletion (Stranger et al., 2005). In addition, ZNRD1 was identified as 1 of the 273 host proteins required for HIV-1 infection and replication in a genome-wide siRNA screen (Brass et al., 2008). An independent study confirmed that the down-regulation of *ZNRD1* through siRNA or shRNA impaired HIV-1 replication at the transcription level (Ballana et al., 2010), suggesting strongly that ZNRD1 is the causal factor for the *ZNRD1*/*RNF39* associations.

In addition to these three loci, all securely replicated associations revealed by GWAS (red in Figure 2) require further functional studies in order to fully understand their causal relationship with HIV control or disease pathogenesis. Potential associations (light blue in Figure 2) require independent replication to support further functional studies.

## Alternative Explorations

Beyond the main GWAS results described above, we believe that other strategies and HIV phenotypes deserve to be mentioned.

Low-frequency SNPs (<5%) are predicted to have larger effect size and contribute to phenotype, but are often overlooked in GWAS because of power. An interesting study re-analyzed data from three European GWAS focusing on these neglected low-frequency SNPs (Le Clerc et al., 2011). The comparison of 365 LTNP with 1,394 HIV-negative controls on 8,584 low-frequency SNPs revealed two independent associations: the *HCP5* rs2395029 signal (*P* = 8.54 × 10^−15^), and the *RICH2* rs2072255 SNP (*P* = 3.30 × 10^−6^). RICH2 is a factor contributing to the externalization of BST-2/Tetherin (Rollason et al., 2009), which prevents HIV-1 virion budding and release (Tokarev et al., 2009). The *RICH2* rs2072255 SNP is in full LD with a coding polymorphism predicted to impact splicing, which could thus alter *RICH2* expression and its subsequent effect on HIV production.

Beyond HIV/AIDS primary incomes, GWAS investigating metabolic, and age-related complications are also emerging: atherosclerosis (Shrestha et al., 2010), chronic liver disease (Rauch et al., 2010), kidney disease (Kopp et al., 2008), neurocognitive disorder (Levine et al., 2012). Understanding the genetic factors contributing to these conditions is essential since they tend to occur more frequently and at an earlier age in HIV-infected subjects compared to the general population.

Finally, a number of alternative strategies have been deployed outside genetics in order to discover some of the host factors involved in viral control, AIDS progression or HIV replication. We can mention transcriptomic (mRNA expression) studies (Giri et al., 2006; Rotger et al., 2010; Salgado et al., 2011; Vigneault et al., 2011), proteomic studies (Coiras et al., 2006; Chan et al., 2007; Molina et al., 2007; Ringrose et al., 2008), or siRNA and shRNA screens (Brass et al., 2008; Konig et al., 2008; Zhou et al., 2008; Yeung et al., 2009). Some of these studies will be further discussed in Santa-Marta et al. (2013).

## Discussion and Future Perspectives

The GWAS focusing on viral control identified *HLA* as the primary region involved in HIV control in both European and African derived populations. Even if the genetic variants highlighted in these two populations were different, it might suggest a similar mechanism of control, engaging notably the antigen presentation by the HLA-B molecule. A role for HLA-C was also unraveled in Europeans through a mechanism involving variation of expression level on the cell surface.

In addition to viral control (and not surprisingly), *HLA* is also the major region involved in long-term non-progression. However, the role for the *HCP5*/HLA-B*57 and −35 kb *HLA-C* SNPs on disease progression was mainly driven by their influence on early viral control. On the other hand, the independent *ZNRD1* signal was associated with disease progression without impacting directly viral replication.

Even if the design of each cohort varies, the contribution of *HLA* variants was consistently replicated in the different studies focusing on viral control and/or disease progression. The differences observed between the studies were mostly due to different genotyping platforms. Indeed, Affymetrix and Illumina genotyping arrays have different human genome coverage: Affymetrix SNPs were selected for their genotyping accuracy and their regular physical spacing across the genome, while Illumina selected SNPs based on the LD (tagSNPs) from the HapMap populations, focused on gene-centric variations, and enriched the coverage in the highly polymorphic *HLA* region. In particular, the difference of coverage for the *HLA* region explains why the cohorts that were genotyped with the Affymetrix arrays (MACS and other natural history USA cohorts (Herbeck et al., 2010; Troyer et al., 2011) did not identify the *HLA* and *ZNRD1* signals. Similarly, the early Illumina 300K chips did not cover the −35 kb *HLA-C* variant, when more dense Illumina chips (≥500 K) do. This explains why this variant was not identify by the ACS, GRIV, and PRIMO studies using Illumina 300K. However, when this variant is genotyped independently, the −35 kb *HLA-C* signal is replicated (van Manen et al., 2009).

Of course, the use of different genotyping arrays is not the only source of variation between the studies. As illustrated by Figures 1, 3, and 4, the cohort size varies across GWAS and this affects statistical power. This power concern might explain why the *HLA* region was not significant in the Hispanic population from the HIV controllers study (International HIV Controllers Study et al., 2010), although we cannot exclude that *HLA* is not involved in viral control in this population.

The exploration of different HIV phenotype is also a great source of variation in the design of all these GWAS. As an example, the examination of a progression phenotype defined according to time to AIDS or to AIDS-related death might explain why *HLA* was not identified as the primary regulator of progression in the ACS cohort (van Manen et al., 2011), although we cannot exclude that a lack of power, a difference of transmission route, or another unknown factor might account for the failure to confirm *HLA*.

Even if it makes it more difficult to compare results among studies, the diversity of phenotype might be advantageous when considering the discovery of the full spectrum of host factors involved in HIV/AIDS pathogenesis. Therefore, the alternative analysis of the GRIV LTNP after excluding individuals controlling viral load at very low levels revealed the importance of *CXCR6* in long-term non-progression (Limou et al., 2010). This association being largely independent of viral control, it was logically not identified by the cohorts designed to specifically investigate viral control (Euro-CHAVI, HIV controllers, etc.). Another striking example of the asset of exploring different endpoints is the GRIV rapid progression GWAS. This underpowered analysis revealed associations with strong effect size (OR from 2.2 to 4.2) and biologically relevant candidate genes (Le Clerc et al., 2009). Rapid progressors are often depleted from cohorts (e.g., enrollment in Euro-CHAVI requires a stable viral load), and thus, the absence of a powerful replication cohort is a severe limitation for further investigation of this unique phenotype. Consequently, it might be very useful for the community to develop new cohorts exploring rapid progressors and other innovative phenotypes in the hope of drawing a complete picture of AIDS pathogenesis.

Genome-wide association studies exploring the HIV acquisition were mainly disappointing since no strong signal outside the *CCR5-Δ32* homozygosity were identified. The lack of significant signals associated with HIV-1 acquisition indicates that common genetic variants with strong effect size are unlikely to play a role in HIV-1 susceptibility in these populations. Three and two GWAS were performed in African and European ancestry populations, respectively. Only the GRIV/ACS European GWAS identified an association with chromosome 8 *CYP7B1* (Limou et al., 2012). The absence of confirmation in the European CHAVI014 Hemophilia cohort might be due to a lack of power (see Figure 4), to a difference of phenotype (GRIV/ACS used normal population controls when CHAVI014 used highly exposed HIV-seronegatives), to a difference of transmission route (GRIV/ACS were mostly infected through sexual contact when CHAVI014 were infected through blood-transfusion), to the identification of a false positive in the GRIV/ACS study, or to an unidentified factor.

Altogether, the *HLA* and chemokine receptors (*CCR2/CCR5* and *CXCR6*) associations explain around 20% of the variance in viral control and/or disease progression, depending on the cohorts. In contrast, most GWAS for common diseases or traits (e.g., type 2 diabetes, height) have identified common variants with small to modest effect sizes explaining a small portion of the phenotypic variance (Lango Allen et al., 2010). With ever larger cohorts, variants with smaller effect sizes might be discovered, but new strategies are also needed to supplement standard genetic analyses.

First, common variants were widely tested in the context of HIV-infected populations of European ancestry. However, we cannot exclude that common variants with small to medium effect size were missed and buried below the very strict statistical threshold (type 2 errors or false negatives) – this is especially relevant in the context of the relatively modest sample size of the HIV/AIDS cohorts. Increasing study power by recruiting additional individuals can contribute to the discovery of new variants associated with HIV. The examination of a bigger sample size for the Euro-CHAVI cohort was indeed successful since it revealed additional *HLA* variants and confirmed the *ZNRD1* signal (Fellay et al., 2009). An alternative solution is to focus on individuals with extreme phenotypes (such as the LTNP, rapid progressors, the highly exposed HIV-seronegatives), who represent a powerful contrasting tool enriched for genetic variants associated with the phenotype. Indeed, the GRIV cohort illustrates the power of a modest size cohort designed with very careful extreme phenotypes to identify new candidate genes (Le Clerc et al., 2009; Limou et al., 2009, 2010). Finally, combining several studies into large meta-analyses also increases power and can thus contribute to the discovery of new host factors. A collaborative effort was initiated in 2009 to create the international consortium on HIV genomics (ICHG) in order to combine the previously published GWAS and explore further the different phenotypes. The first phase of meta-analysis focusing on HIV-1 acquisition by comparing 10,000 HIV-infected subjects with 15,000 HIV-uninfected has been completed and did not identify any new signal beyond the resistance conferred by *CCR5-Δ32* homozygotes, suggesting again that common host genetic variation has little influence on HIV susceptibility (McLaren et al., 2012b). The second phase of ICHG meta-analysis is on-going and will focus on phenotypes associated with viral control and disease progression.

African populations have also been explored during this first wave of GWAS, but not for the analysis of disease progression phenotypes. This oversight stems from funding and infrastructure limitations to support large prospective cohorts. In addition, African populations exhibit a higher genetic diversity and a lower LD level than populations outside of Africa, and therefore require arrays with a higher density of tagSNPs to ensure a genome-wide capture. The new generation of genotyping arrays now offers the simultaneous genotyping of up to 5 M tagSNPs, and incorporates the knowledge from the 1000 Genomes Project (new public catalog of human genetic variants with a frequency down to 1%) (1000 Genomes Project Consortium et al., 2012). Even if the coverage is still less effective for African populations than for European and Asian populations, the use of these new genotyping arrays might participate in the discovery of common and less common genetic variants associated with HIV-related outcomes in Africans.

Other HIV-infected populations with different ancestries have been widely disregarded in the host genetics field (e.g., Asian populations, admixed-populations such as Latinos and Hispanics). These populations may harbor different variants from Europeans that also influence HIV infection, replication, and pathogenesis.

Beyond the common polymorphisms (with a frequency >5%) targeted during the first wave of GWAS, rare SNPs, indels, and structural variants might also contribute to HIV infection and AIDS progression. The new generation of genotyping arrays encompasses information for lower frequency SNPs (1–5%) from the 1000 Genomes Project. The use of this newer high density arrays combined with the next-generation sequencing (exome or genome) should capture rare SNPs, indels, and structural variants, and highlight new host factors associated with HIV infection course. Since next-generation sequencing is an expensive technology, focusing on extreme phenotypes to emphasize the genetic differences as in the vanguard GRIV cohort (Hendel et al., 1996) should prove a promising strategy (McLaren et al., 2011; Pelak et al., 2011).

The next challenges are to assess the computationally challenging gene × gene and gene × environment interactions, as well as the role of epigenetics in HIV disease. The incorporation of biological knowledge might aide in assessing these complex interplays under reasonable assumptions. Systems biology integrates information from multiple “omics” sources (genomics, transcriptomics, proteomics, etc.), and could thus disclose specific host pathways or networks contributing to the HIV-linked phenotypes.

Finally, we cannot forget that HIV course is also impacted by viral and environmental factors that may play a larger role than initially suspected (Wang, 2013).

## Conclusion

The motivation for identifying genes associated with HIV/AIDS has gained momentum as effective vaccines and curative treatments have failed to materialize. The technologic breakthrough of genotyping arrays has opened up a prolific era in human genetics. Relatively to the discovery of hundreds of genes involved in dozen of complex diseases, the re-discovery of *HLA* and chemokine region in the HIV/AIDS field could seem quite disappointing. However, it is important to note that GWAS revealed that *HLA* is the primary region involved in viral control. In addition, two signals were discovered (*CXCR6* and *PARD3B*), and candidate regions highlighted. New strategies to exploit the large amount of genomic data generated (e.g., meta-analyses, systems biology, etc.) and the generation of new data (e.g., new phenotypes, new populations, next-generation sequencing, etc.) will hopefully lead to the discovery of new host genetic associations that could lead, following the example of *CCR5-Δ32*, to the development of new antiretroviral therapy or vaccine.

## Conflict of Interest Statement

The authors declare that the research was conducted in the absence of any commercial or financial relationships that could be construed as a potential conflict of interest.

## Abbreviations

**Genes:** *CCR2*, C-C chemokine receptor 2; *CCR5*, C-C chemokine receptor 5; *CXCR6*, C-X-C chemokine receptor 6; *CYP7B1*, cytochrome P450, family 7, subfamily B, polypeptide 1; *HCP5*, HLA complex P5; *HLA*, human leukocyte antigen; *KLF3*, Kruppel-like factor 3; *PARD3B*, PAR-3 partitioning defective 3 homolog B; *PRMT6*, protein arginine methyltransferase 6; *PROX1*, prospero homeobox 1; *RNF39*, ring finger protein 39; *RXRG*, retinoid X receptor gamma; *SOX5*, SRY-box 5; *TBC1D1*, TBC1 domain family member 1; *TGFBRAP1*, transforming growth factor beta receptor associated protein 1; *ZNRD1*, zinc ribbon domain containing 1.

**Cohorts:** ACS, Amsterdam cohort study; ALIVE, AIDS link to intravenous experience; CHAVI, center for HIV/AIDS vaccine immunology; COS, Couples Observational Study; DCG, DC gay; DoD HIV NHS, Department of Defense HIV Natural History Study; GISHEAL, genetic and immunological study on HIV^+^ European and African LTNP; GRIV, genomics of resistance to immunodeficiency virus; HGDS, Hemophilia Growth and Development Study; LSOCA, longitudinal studies of the ocular complications of AIDS; MACS, multicenter AIDS cohort study; MHCS, multicenter Hemophilia cohort study; PPHHTS, Partners in Prevention HSV/HIV Transmission Study; PRIMO, primo infection; SFCC, San Francisco city clinic.
